# Children’s Teeth

**Published:** 1891-08

**Authors:** 


### CHILDREN'S TEETH.

"Let good digestion wait on appetite and health on both," says Shakespeare. Health will fail to "wait" on either if parents will allow their own and their children's teeth "to become a mass of decay" at an early age.

There is no one point on which people are so careless as the proper care of children's first teeth, and those of the second set that erupt between the fifth and seventh years of age.

There is no one condition that tends in a greater degree to produce good health and vigorous growth of the body than a good sound set of teeth.

People do not relish the idea of being told they are careless or worse, but it would seem that a subject of such vital importance would receive the most careful attention.

Incalculable harm is done to both the health of a child, and to the integrity of the second set of teeth, in allowing the temporary teeth to become decayed and abscessed, carrying pain and suffering and frequently indigestion and all its accumulated evils.

The number of children who have decayed teeth, and in many cases, a part of the first set gone and the second set badly broken down, is too great.

"Neglect is the mortal enemy of the teeth." If the first set of teeth is lost before the proper time, the second sets suffer much from their loss, and in some cases does not erupt at all. If decayed, the first should be filled with plastic filling material and let remain until their places are ready to be taken by the second teeth.

But a great deal of good can be accomplished by keeping the teeth brushed and cleaned. The patient should be taught to brush his own teeth and use the pick after every meal. It will do the parent no harm to practice the lesson occasionally himself.

In this manner one can save more teeth, using no instruments but the brush and pick (and, by the way, one should use nothing but a quill tooth pick) and silk thread, than all the dentists can by performing their usual dental operations.

It must not be inferred that we can, by any means, always or in every case avoid the necessity of filling children's teeth. But when cared for properly the defect would be detected at so early a stage that the operation for repair (filling) would be painless, not tedious, involving but little expense. and its durability beyond question.

If not filled then, while decaying, the mouth will be foul and unhealthy, the lips and tongue will be irritated, often severely, by the rough and ragged edge presented, the decay will be likely to reach the pulp, causing excruciating pain, the death and premature loss of the tooth and lasting injury to the jaws and position of the incoming set.

The child will not and cannot chew on sore gums and teeth. The food will be put down and out of the way as soon as possible without the proper preparation of it for the stomach, and the result is early dyspepsia with its train of horrors. The one point of paramount importance which I wish to urge is, that the teeth should be kept clean from their first appearance through the gums, no matter how young the child may be, even if born with teeth, as some are.

They should be kept as scrupulously clean as the cheeks, the eyes, or the ears, for they will suffer more from neglect, even though milk be the only food for the younger years. The brush is the only thing that will accomplish this.—*The Healthy Home.*

